# The Efficacy of Herbal Supplements and Nutraceuticals for Prevention of Migraine: Can They Help?

**DOI:** 10.7759/cureus.14868

**Published:** 2021-05-06

**Authors:** Kavaljeet Kaur, Vernicia Hernandez, Sari W Al Hajaj, Ahmed M Ebrahim, Mirash Razack, Mohamed W ElSharief, David Dragas

**Affiliations:** 1 Internal Medicine, California Institute of Behavioral Neurosciences & Psychology, Fairfield, USA; 2 Surgery, California Institute of Behavioral Neurosciences & Psychology, Fairfield, USA; 3 Emergency Medicine, California Institute of Behavioral Neurosciences & Psychology, Fairfield, USA; 4 Medicine, Wuhan University, Wuhan, CHN; 5 Internal Medicine, Al Ain Hospital, Al Ain, ARE; 6 Pediatrics and Child Health, California Institute of Behavioral Neurosciences & Psychology, Fairfield, USA; 7 Research, California Institute of Behavioral Neurosciences & Psychology, Fairfield, USA

**Keywords:** migraine, headache, nutraceuticals, antioxidants, coenzyme q10, herbal supplements, prevention, riboflavin

## Abstract

Migraine is a common neurological disorder associated with or without aura. Although the pathophysiology of migraine is not very well understood, pro-inflammatory cytokines and oxidative stress biomarkers are found to be increased in migraine. Multiple studies have been done to see if alternative medicine such as herbal supplements and nutraceuticals are effective in the prevention and treatment of migraine headaches. This review aimed to evaluate the effect of supplements like coenzyme Q10, riboflavin (vitamin B2), feverfew, and magnesium on the frequency, severity, and duration of migraine attacks.

We performed a thorough literature search using mainly PubMed. We included studies published in the last 10 years, those conducted among adult human participants 18-65 years of age, and those published in the English language. Based on the articles selected for the final review, we concluded that herbal supplements and nutraceuticals help reduce the frequency of migraine headaches; however, mixed results were seen regarding the severity and duration of headaches. Moreover, there were no concerning side effects with these supplements. Therefore, physicians can suggest herbal supplements to patients who experience adverse effects from pharmaceutical drugs and desire a more natural treatment.

## Introduction and background

Migraine is characterized by moderate-to-severe episodic, throbbing, unilateral headaches usually accompanied by nausea, vomiting, photophobia, and phonophobia [1]. According to the Global Burden of Disease report from the World Health Organization, it is the third most prevalent and the sixth most debilitating disease globally. Migraine is three times more common in females than in males with a worldwide prevalence of 14.7% [2]. Overall, 2% of the world’s population and, on average, one in seven Americans suffer from migraines [3,4].

The pathophysiology of migraine is unclear. Studies with phosphorus magnetic resonance spectroscopy (31P-MRS) show altered energy metabolism in the brain. These abnormalities in energy metabolism seem to have a genetic component and a neurovascular component. Migraine is associated with a mutation in genes coding for metabolic enzymes in both mitochondrial and nuclear DNA [5-7]. The neuronal and vascular dysfunction observed can be explained by impaired oxygen metabolism resulting from mitochondrial dysfunction, oxidative stress, and inflammation resulting in changes in vascular tone [8,9]. Migraine is associated with other disorders such as cardiovascular disease, stroke, asthma, epilepsy, allergies, and sleep disorders [10]. It is usually triggered by fasting, skipping meals, stress, and lack of sleep [11,12].

Migraine is a chronic debilitating condition impacting daily life. Proper treatment and prevention can reduce the burden on healthcare and improve the quality of life of patients. Acute attacks are treated with analgesics or triptans. Drugs used for prophylaxis include anti-depressants (amitriptyline), anti-epileptics (topiramate, valproic acid), beta-blockers (propranolol), and calcium-channel blockers.

Recently, the use of herbal supplements and nutraceuticals such as coenzyme Q10, vitamin B2 (riboflavin), feverfew, and magnesium seem to be gaining popularity for the prevention of migraine. One of the reasons for their rising popularity is fewer side effects in comparison to pharmacological drug therapy. With this review article, we intend to explore the effectiveness of nutraceuticals and herbal supplements for the prevention of migraine attacks. We expect that this article will help guide physicians to decide which preventive treatment suit a patient better and thus help with future migraine attacks.

## Review

We performed a thorough literature search using PubMed to identify relevant studies. Studies published in the last 10 years, performed among human adult populations 18-65 years of age, and published in the English language were included. The search was done using keywords “migraine,” “nutraceuticals,” “supplements,” “Coenzyme Q10 ubiquinone,” and “prevention and control.”

The initial search yielded 106 articles, but after careful screening, applying inclusion and exclusion criteria, and excluding duplicate articles, 36 were shortlisted for abstract and full-text review. These included randomized controlled trials, observational studies, cohort studies, systematic reviews, and meta-analysis. Animal studies, case reports, and studies on pediatric and adolescent populations were excluded.

A total of 36 studies selected for the final review included two systematic reviews with meta-analysis, four randomized placebo-controlled double-blinded trials, three open-label pilot studies, one observational study, one cohort study, and review articles. Table 1 presents the studies and their findings.

**Table 1:** Effect of nutraceuticals on migraine headache. TNF-α: tumor necrosis factor-α; CGRP: calcitonin gene-related peptide; IL-6: interleukin-6; IL-10: interleukin-10; HIT-6: headache impact test; HDR: headache diary results

Author/Year	Type of study	No. of patients	Purpose of study	Conclusion/Result
Dahri et al. 2018 [13]	Randomized double-blind placebo-controlled clinical trial	52	Effect of coenzyme Q10 on the clinical features of migraine and inflammatory markers	Significant reduction in TNF-α and CGRP, no significant difference in serum IL-6 and IL-10 levels
Gaul et al. 2015 [14]	Randomized double-blind placebo-controlled clinical trial	130	Effect of coenzyme Q10, riboflavin, and magnesium on the clinical features of migraine	Significant reduction in the intensity of migraine pain (p = 0.03) and score of the HIT-6 questionnaire (p = 0.01)
Shoeibi et al. 2016 [15]	Open-label parallel add-on, controlled trial	80	Effect of coenzyme Q10 in the prevention of migraine	Significant reduction in the frequency and intensity of migraine (p < 0.001)
Guilbot et al. 2017 [16]	Prospective observational	132	Effect of coenzyme Q10, magnesium, and feverfew in the prevention of migraine	Significant reduction in the frequency of migraine attacks (1.3 days ±1.5 vs. 4.9 days ±2.6; p < 0.0001)
Parohan et al. 2019 [17]	Systematic review and meta-analysis	221	Effect of coenzyme Q10 in the prevention of migraine	Significant reduction in the frequency of migraine attacks (p < 0.0001), no significant reduction in severity and duration
Hajihashemi et al. 2019 [18]	Randomized double-blind placebo-controlled clinical trial	56	Effect of coenzyme Q10 and L-carnitine supplementation in the prevention of migraine	Significant reduction in serum lactate level (p < 0.0001), frequency, severity, and duration of migraine attacks (p < 0.0001)
Volta et al. 2015 [19]	Pilot study	40	Effect of coenzyme Q10 on episodic migraine without aura	Reduction in both frequency and intensity were seen in 25 patients, reduction in intensity but not in frequency in 10 patients, and no reduction in either in 5 patients
Sazali et al. 2021 [20]	Systematic review and meta-analysis	371	Effect of coenzyme Q10 in the prevention of migraine	Significant reduction in the frequency and duration of migraine attacks (p < 0.0001), no significant reduction in severity
Parohan et al. 2019 [21]	Randomized double-blind placebo-controlled clinical trial	100	Effect of nano-curcumin and coenzyme Q10 supplementation in the prevention of migraine	Significant reduction in the frequency, severity, and duration of migraine attacks and HDR (for all p < 0.001)
Vikelis et al. 2020 [22]	Pilot single-arm open-label	113	Effect of coenzyme Q10, riboflavin, magnesium, feverfew, and panniculata on the clinical features of migraine	Significant reduction in frequency, days with peak intensity headache, and HIT-6 scores (p < 0.001)
Pucci et al. 2015 [23]	Cohort	20	Effect of coenzyme Q10 in the prevention of migraine	Significant reduction in the frequency of migraine attacks

Discussion

**Figure 1:**
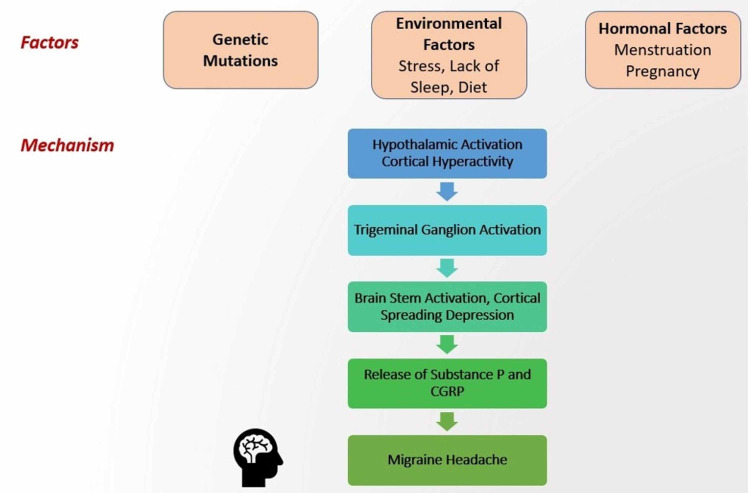
Pathophysiology of migraine headache. CGRP: calcitonin gene-related peptide

Migraine is considered to occur due to disturbances in brain homeostasis. The pathophysiology of migraine is unclear but seems to include a genetic component and a neurovascular component. Mutations in chromosome 19 are seen to be linked with familial migraine. Mutations involving Cav2.1 (P/Q)-type voltage-gated calcium channel *CACNA1A* gene are seen in about 50% cases of familial migraine [24]. One consequence of this mutation may be increased glutamate release. Mutations in the *ATP1A2* gene have been identified to be responsible for about 20% of familial cases. *ATP1A2* gene codes for a Na+/K+ ATPase. Mutation in this gene leads to decreased activity of astrocytic glutamate transporters and eventually build-up of synaptic glutamate. The increased glutamate seems to cause migraine headaches [25]. Figure 1 shows the various triggers and pathophysiology of migraine headache.

The occurrence of migraine is associated with cortical hyperactivity. The trigeminal ganglion is hypothesized to be involved in this hyperactivity [26]. Activation of trigeminal sensory nerve fibers, in turn, causes brainstem activation and induces the release of vasoactive peptides such as substance P and calcitonin gene-related peptide (CGRP). This causes platelet and endothelial activation and, consequently, an increase in nitric oxide synthesis, vasodilation, leakage of blood vessels, and degranulation of mast cells [27]. This further activates sensory trigeminal fibers, increasing the release of substance P and CGRP, and transmits pain impulses throughout the brain [28].

Migraine with aura is associated with sensory, visual, or motor disturbances. These include increased sensitivity to light or sound, zig-zag flashes of light, and numbness or tingling in one hand or face. It is seen in about 30% of patients. Aura is considered to occur due to the slow wave of depolarization spreading across the cortex. This leads to stimulation of the trigeminovascular system, cerebral vessels nociceptors, and regions of the brain responsible for triggering pain and other neurological symptoms [29].

Studies with 31P-MRS have shown altered energy metabolism in the brain during acute migraine attacks. Hence, migraine can be triggered by fasting, physical exertion, excess sleep or sleep deprivation, intense aromas, or photosensitivity, which cause impaired oxygen metabolism due to mitochondrial dysfunction, oxidative stress, and inflammation [8,9]. Several oxidative stress biomarkers are found to have increased plasma levels in patients with migraine. Table 2 presents studies showing the association of migraine with inflammation and oxidative stress.

**Table 2:** Association of migraine with inflammation and oxidative stress. TAS: total antioxidant status; TOS, total oxidant status; OSI: oxidative stress index; 8-OHdG: 8-hydroxydeoxyguanosine; MDA: malondialdehyde; UTS2R: urotensin 2 receptor; CAT: catalase; WHM: white matter hyperintensities

Author	No. of patients	Migraine type	Results
Alp et al. [30]	75	Without aura	Decrease in TAS; increase in TOS; increase in OSI during the attack-free period
Geyik et al. [31]	50	With and without aura	No differences in TOS, TAS, and OSI in migraine patients with and without aura. Increase in 8-OHdG in migraine patients without aura versus with aura
Yigit et al. [32]	40	Without aura	Increase in lymphocyte DNA damage. Elevated values of TOS, OSI, and MDA and decreased value of TAS in migraineurs. Decreased plasma UTS2R in migraineurs. Decreased CAT activity in migraineurs
Aytaç et al. [33]	32 (18 with WHM and 14 without WHM-type lesions)	With and without WHM	Decreased CAT activity and increased MDA level in migraine patients with WHM-type lesions versus without WHM-type lesions
Tozzi-Ciancarelli et al. [34]	23	With aura	Increase in concentration of substances reacting with thio-barbituric acid during attack-free periods

Due to their antioxidant properties and involvement in the mitochondrial electron transport chain, many nutraceuticals and herbal supplements such as coenzyme Q10, vitamin B2 (riboflavin), magnesium, alpha-lipoic acid, vitamin C, curcumin, and feverfew are considered for prophylaxis of migraine.

Coenzyme Q10, or ubiquinone, is a natural lipophilic substance needed for all cellular processes requiring energy. It acts as the complex III component of the electron transport chain and freely moves throughout the inner mitochondrial membrane transferring electrons from complex I and complex II (NADH dehydrogenase and succinate-Q-reductase, respectively) to cytochrome C. Coenzyme Q10 has been hypothesized to be useful in migraine prevention because of its vital role in sustaining mitochondrial energy stores [35]. In addition to its actions as an electron carrier, it also exerts an anti-inflammatory effect via nuclear factor kappa B signaling pathway inhibition preventing excessive reactive oxygen species production and inhibiting lipid membrane peroxidation and nucleic acid oxidation [35,36]. This helps with the inflammatory component of migraine. Recently, it is seen that coenzyme Q10 administered to migraine patients results in a decrease of CGRP, which is an important vasoactive peptide involved in the pathogenesis of migraine [13].

Curcumin has also been studied for the treatment of migraine. It is a lipophilic substance and acts as an anti-oxidant. It decreases the production of reactive oxygen species and inflammatory mediators such as interleukins (IL-1, IL-6) and cyclooxygenase [21].

Vitamin B2 (riboflavin) is a precursor for flavin-mononucleotide and flavin adenine-dinucleotide involved in the electron transport chain in the mitochondrial membrane. It is considered useful in migraine prevention due to its role in maintaining the energy stores of the body.

Magnesium (Mg) is involved in various biological processes. It acts as a cofactor for ATP-synthase which is involved in the production of ATP. Furthermore, it also regulates neuronal excitability and plays an important role in the regulation of vascular tone [37].

Feverfew (*Tanacetum parthenium*) is also thought to be helpful in migraine [38,39]. Since ancient days, it has been used for pain, inflammation, nausea, and vomiting [40]. It is largely found in South America. Its main active component is parthenolide which inhibits aldose reductase activity and causes relaxation of vascular smooth muscle. It also exhibits anti-inflammatory effects [41,42], all of which contribute to its anti-migraine effects.

The most common side-effects of magnesium and feverfew are limited to the gastrointestinal system and include nausea, diarrhea, appetite suppression, heartburn, and epigastric discomfort with an incidence of less than 1% [43,44].

Guilbot et al. conducted a prospective observational study on 132 adults, mainly women (91.2%), in 2017 on the effect of a combination of coenzyme Q10, feverfew, and magnesium on the prevention of migraine. Supplementation significantly reduced the frequency of migraine attacks by the third month without any significant reduction in intensity. Overall, 75% of patients showed a reduction of at least 50% in the number of days with migraine headache per month [16].

Another study conducted by Gaul et al. in 2015 on the effect of coenzyme Q10, riboflavin, and magnesium supplementation on the clinical features of migraine included 130 adult migraineurs (age 18-65 years) with more than three migraine attacks per month. Patients were randomized in the treatment group and placebo group in a double-blinded manner and followed for three months. The frequency of headaches was reduced in the treatment group. The intensity of headache was also significantly reduced in the treatment group (p = 0.03). The sum score of the HIT-6 (headache impact test) questionnaire was reduced in the treatment group [14].

Dahri et al. conducted a randomized, double-blind, placebo-controlled clinical trial in 2018 among 45 women aged 18-50 years to assess the effect of coenzyme Q10 on the clinical features and inflammatory markers of migraine. The treatment group received 400 mg/day coenzyme Q10 for three months. Significant reduction in CGRP and TNF-α levels was seen (p = 0.011 and p = 0.044, respectively), but no significant difference in serum IL-6 and IL-10 was reported. A significant increase in coenzyme Q10 level (P < 0.001) and reduction in the frequency, severity, and duration of migraine attacks was reported in the treatment group [13].

Two systematic reviews and meta-analyses on the effect of coenzyme Q10 in the prevention of migraine included 592 patients. Both studies concluded a significant reduction in the frequency of migraine attacks (p < 0.0001) but no significant reduction in severity [17,20].

A cohort study among 20 patients, aged 22-49 years, with a male/female ratio of 1:5 by Pucci et al. showed a significant reduction in the frequency of migraine attacks on supplementation of coenzyme Q10 [23].

Two randomized, double-blind, placebo-controlled clinical trials including 156 patients showed a significant reduction in the frequency, severity, and duration of migraine attacks (p < 0.001) when coenzyme Q10 was supplemented with other supplements such as L-carnitine and nano-curcumin [18,21].

Shoeibi et al. conducted an open-label, parallel, add-on, controlled trial on the effect of coenzyme Q10 in the prevention of migraine. A total of 80 patients were allocated to receiving only their current preventive drugs or their current preventive drugs plus 100 mg coenzyme Q10 daily for three months. A significant reduction was evident in the frequency (1.6 vs. 0.5; p < 0.001) and severity of headaches (2.3 vs. 0.6; p < 0.001) among coenzyme Q10 and control groups [15]. Other pilot studies also showed a reduction in the frequency of migraine attacks with coenzyme Q10 supplementation [19,22].

Limitations

We did not include any animal studies, case reports, and studies on the pediatric and adolescent populations. Moreover, we excluded studies published in other than the English language which may have impacted the study findings.

## Conclusions

Studies discussed in this review confirm the involvement of oxidative stress in migraine, and therefore, antioxidants seem to be helpful in its prevention. Based on our research, several studies showed that vitamins and minerals including coenzyme Q10, magnesium, riboflavin, and feverfew reduced the number of days of migraine attacks in patients. Few studies also showed a decrease in the intensity and duration of migraine headaches. However, more research is needed to conclude the effects of herbal supplements on the prevention of migraine headaches. Because many migraine triggers are our day-to-day activities, physicians should educate their patients about lifestyle modification and suggest herbal supplements, vitamins, and minerals to patients who experience side effects from pharmaceutical drugs and desire a more natural treatment.
